# The Gold Inlay Not Easily Made

**Published:** 1918-09

**Authors:** J. J. Travis


### THE GOLD INLAY NOT EASILY MADE

BY J. J. TRAVIS, D.D.S.

The men who attempt to do the gold inlay because it is an easy method, are making a mistake. It is not easy. It is one of the hardest pieces of work it is possible to turn our hands to, and requires a greater knowledge of the procedure than does the malleted gold filling. You must be familiar with the action of your wax and the volume changes. You must be familiar with the temperature at which the investment is to be made; with the consistency of the investment and the volume changes (shrinkage or expansion) that would result from the different methods of handling it. You must be familiar with the percentage of the flare of the wall because of the shrinkage of the gold, and when you attempt to have the shrinkage of the gold counterbalanced by the expansion of the wax, or the expansion of the investment, the temperature at which you shoot your gold into the investment and the difference in shape of cavity preparation—which determines the temperature at which you should cast—these things must all be known and worked out scientifically.

No man can get ideal results with the gold inlay and call it an easy process. It is easier on the patient, and that is an advantage. It is somewhat easier on the nervous system of the operator, because he is not confined at the operation so long at any time. To do a really careful operation in filling a tooth with a cemented gold inlay, is one of the most difficult operations we have to perform, and the sooner the profession gets rid of the idea that this is an easy operation the sooner we will get to making gold inlays that will present to us the margin of the malleted gold filling, not only on exposed and accessible surfaces, but at the cervical margin.—*Journal N. D. A.*, August, 1918.

